# Word frequency effects found in free recall are rather due to Bayesian surprise

**DOI:** 10.3389/fpsyg.2022.940950

**Published:** 2022-08-25

**Authors:** Serban C. Musca, Anthony Chemero

**Affiliations:** ^1^Department of Psychology, Université Rennes 2, Rennes, France; ^2^Department of Philosophy and Psychology, University of Cincinnati, Cincinnati, OH, United States

**Keywords:** word frequency, free recall, ecological psychology, predictive coding, Zipf’s law, Bayesian surprise, mixed lists, pure lists

## Abstract

The inconsistent relation between word frequency and free recall performance (sometimes a positive one, sometimes a negative one, and sometimes no relation) and the non-monotonic relation found between the two cannot all be explained by current theories. We propose a theoretical framework that can explain all extant results. Based on an ecological psychology analysis of the free recall situation in terms of environmental and informational resources available to the participants, we propose that because participants’ cognitive system has been shaped by their native language, free recall performance is best understood as the end result of relational properties that preexist the experimental situation and of the way the words from the experimental list interact with those. In addition to this, we borrow from predictive coding theory the idea that the brain constantly predicts “what is coming next” so that it is mainly prediction errors that will propagate information forward. Our ecological psychology analysis indicates there will be “prediction errors” because the word frequency distribution in an experimental word list is inevitably different from the particular Zipf’s law distribution of the words in the language that shaped participants’ brains. We further propose the particular distributional discrepancies inherent to a given word list will trigger, as a function of the words that are included in the list, their order, and of the words that are absent from the list, a surprisal signal in the brain, something that is isomorphic to the concept of Bayesian surprise. The precise moment when Bayesian surprise is triggered will determine to what word of the list that Bayesian surprise will be associated with, and the word the Bayesian surprise will be associated with will benefit from it and become more memorable as a direct function of the magnitude of the surprisal. Two experiments are presented that show a proxy of Bayesian surprise explains the free recall performance and that no effect of word frequency is found above and beyond the effect of that proxy variable. We then discuss how our view can account for all data extant in the literature on the effect of word frequency on free recall.

## Introduction

Contradictory results have been reported in the literature that examines the effect of the frequency of occurrence of words in the language (hereafter, word frequency, WF) on free recall (hereafter, FR). It first appeared that WF was positively related to FR performance, with high frequency (HF) words having a higher probability of being recalled than low frequency (LF) words (e.g., Hall, 1954; Murdock, 1960; Sumby, 1963). However, it was found the experimental design used influences the results, with different effects that arise from the manipulation of WF between-subjects (pure lists: a participant either receives only LF words or only HF words) vs. within subjects (mixed list: all participants receive a list comprising words of all frequencies). FR performance of mixed lists has been found to be negatively related to WF in some studies (DeLosh and McDaniel, 1996; Merritt et al., 2006; Ozubko and Joordens, 2007), but sometimes no relationship was found (Watkins et al., 2000; Ward et al., 2003; Ozubko and Joordens, 2007), and other studies found a positive relationship between WF and FR performance (Balota and Neely, 1980; Watkins et al., 2000; Hicks et al., 2005).

Tentative explanations go back to the 1970s (e.g., Brown et al., 1977; see also Glanzer and Adams, 1985) but recent work (Lohnas and Kahana, 2013) indicates that a consensus has not yet been reached. Lohnas and Kahana (2013) observed that the frequency values of “LF” and of the “HF” word list varied greatly between different studies. This prompted them to conjecture a non-monotonic relationship between WF and probability of recall, so Lohnas and Kahana (2013) proposed that a non-monotonic relationship of the kind conjectured would have the potential to reconcile the extant divergent results. Their experimental results do indeed show a non-monotonic relationship between WF and FR performance. Having partitioned the word pool into ten log frequency bins ranging from LF to HF, Lohnas and Kahana (2013) found the probability of FR as a function of the frequency bin to have the shape of a check mark: it was high for the first bin (i.e., the bin of lowest word frequencies), then plummeted to its lowest value for the second bin — to a value significantly lower than that for the first bin — and increased from the third bin on, reaching its peak around the tenth bin (i.e., the bin of the highest frequencies considered). While this result pattern helps to make sense of the contradictory results found in the literature, it does raise the question of why WF has such a peculiar non-monotonic effect on recall performance. This could lead one to suspect the observed effect is not a genuine effect of WF, or that some other variable is also at play. On the other hand, if it is indeed a genuine WF effect, the non-monotonic relationship found poses a challenge to extant models, all of which, to our best knowledge, posit a monotonic relationship between WF and FR.

Explanations exist for the negative and for the positive relationship between WF and recall, but no explanation encompasses both. A decreasing FR performance as WF increases (i.e., a *negative* relationship) can be explained by proposing that “recall performance results from the relative contributions of individual-item information and serial-order information” and that for “common items [i.e., HF words], order encoding should decrease from pure lists to mixed lists [while the] reverse should occur for unusual items [i.e., LF words]: order encoding should increase from pure to mixed lists” (DeLosh and McDaniel, 1996; Merritt et al., 2006). Ozubko and Joordens (2007) explain a negative relation by the asymmetrically strong links between low- and high-frequency words, with high-low intra-list associations being stronger (than the low-high ones) and thus giving rise to better performance on low-frequency words in the mixed list with random word order.

Increased FR performance as WF increases (i.e., a *positive* relationship) is accounted for by explanations originating from the two-process (generate-recognize) theories of recall and recognition (Kintsch, 1968; Anderson and Bower, 1972): HF words are easier to recall because more experience with HF words makes it easier to generate them as retrieval candidates than LF words. In particular Balota and Neely (1980) state that “this can be accommodated by [generate-recognize theories] by arguing that the nodes corresponding to HF words are much more likely to be generated than are nodes corresponding to LF words. Thus, even though it may be more difficult to recognize a generated node corresponding to an HF word than to recognize a generated node corresponding to an LF word, there are so many more HF than LF nodes generated that the net result is superior RCL [recall] of HF words.” A different explanation (Gillund and Shiffrin, 1984) starts from the proposal that HF words have stronger associative relations to other items than do LF words, both in terms of prior associations and in terms of experimental associations formed during the study. Because HF words have stronger associations than LF words, pure HF lists are more easily recalled than pure LF lists. Moreover, as LF words in mixed lists are cued by HF words, there is a better recall of LF words in mixed lists as compared to pure LF lists (which would explain why often no WF effect is found in mixed lists). Finally, because LF words are less effective cues than HF words, HF words are easier to recall in pure HF lists than in mixed lists.

Regardless of the ongoing debate on the merit of these explanations (and others not mentioned here), one is still free to pick the theoretical explanation of the WF effect that fits their results, and no particular theory can explain a non-monotonic relationship, such as the one found by Lohnas and Kahana (2013).

The ideas that we propose here aim at explaining all the result patterns found in the relationship between WF and FR: positive and negative relations, and also no relation, for mixed lists; a consistent positive relation for pure lists. Our tentative explanation will also speak to why the use of pure lists increases the probability of finding a WF effect on FR (as opposed to when a mixed list is used). Our claims are the following. Firstly, there are inevitable discrepancies that exist between the WF distribution of those-words-in-that-experimental-list and of the words in participants’ native language. Secondly, people’s brains are sensitive to such distributional discrepancies, such that Bayesian surprise (Itti and Baldi, 2009; Baldi and Itti, 2010) occurs, the result of which is that some words become more memorable than others. These claims offer a very different account of the data, one grounded in an ecological psychology approach to cognition in general, and in an ecological psychology analysis of the experimental situation used to derive FR performance. We present two behavioral experiments to support our account, and discuss the value of our interpretation with respect to the results of these experiments but also based on how our account may also explain when and why WF effects on FR performance have been found in the literature.

### Inevitable distributional discrepancies between those-words-in-that-experimental-list and the words in participants’ native language

It is central to our main claims that the WF distribution of words in an experimental list is inevitably different from the WF distribution of words in the language. We will thus make this point here and consider its implications.

**FIGURE 1:**
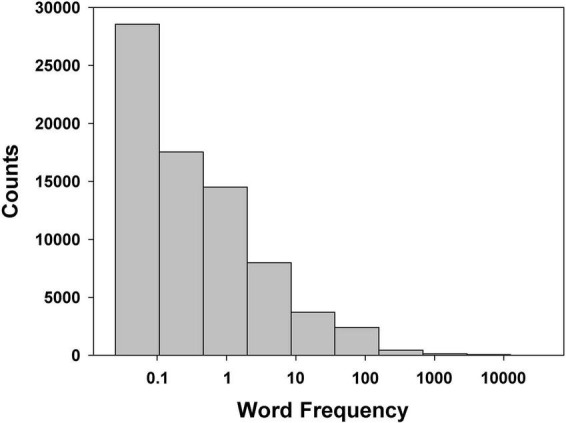
Zipfian distribution of words in American English: count of words per bin (all words in American English are considered for this example). Bins are created by partitioning the frequency range into ten intervals of equal log frequency width. The abscissa represents occurrences of a word per one million words (log scale). Data is based on the SUBTLEX-US database (Brysbaert and New, 2009).

Zipf (1935) showed that WF distribution in English^1^ is very skewed and right-tailed, meaning that a word’s frequency of use is inversely proportional to its rank frequency (see Figure 1) — a distribution later referred to as Zipfian. We like to think Zipf’s line of research did not have the influence it deserves because to our best knowledge no researcher tried to make sure the WF distribution of the words in their experimental list(s) of words was similar to that in participant’s native language or to explain the WF effect(s) on FR as arising from the discrepancies between these two distributions. Of course, one possibility is that those distributional discrepancies do not matter. Not only do we suggest they do, but we will bring some arguments that suggest this is how WF exerts its effect on FR performance.

**FIGURE 2:**
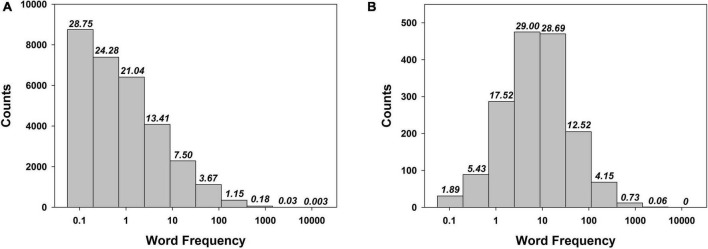
Count of words per bin (and percentage out of a total per bin, italicized, on top of each bar): for all English nouns in the CELEX 2 database **(A)** vs. for the words used by Lohnas and Kahana (2013) in their experiment **(B)**. Note the massive shape difference between the two distributions. The abscissa represents occurrences of a word per one million words (log scale). See text for details.

Figure 2 displays a visual comparison between the WF distribution of all English nouns in the singular in the CELEX 2 database (Baayen et al., 1995), in pane a, and of the English nouns in singular used by Lohnas and Kahana (2013) in their experiment, in pane b. A visual inspection reveals what further analyses confirm. Firstly, the distribution of our reference population of words (pane a), follows a Zipfian distribution, that is, the relation between the ranks of the words and their frequency of use follows a decaying exponential law — a decaying exponential model explains 96.1% of the variance in the data (*p* < 0.0001). Secondly, the distribution of the experimental word pool shown in the pane b of Figure 2, does not follow a Zipfian law: a decaying exponential law fits poorly the data, explaining only 25.34% of the variance (*p* > 0.14) — actually, the distribution is not different from a Gaussian distribution (W = 0.8966, *p* > 0.2). An exact multinomial test, carried out under a model comparison approach based on the calculation of the Bayes factor (BF), confirmed the difference between the two distributions — it yielded a posterior probability of virtually one for the model supposing the two distributions are different, BF_10_ > 10^99^.

One could argue there is no way to warrant that other published studies did not use a WF distribution not too different from a Zipfian one. It is important to comprehend this *cannot* be the case. Indeed, if one used in their experimental list just one noun from the three bins of highest WF, they should also include about 139 (!) words from the bin of the lowest WF (and about 117 from the next bin, and so on), which make for quite an unpractical experimental list. The problem is even more acute when one considers not the pool of all experimental words that were used (as we considered in our example here) but the specific composition of an experimental list of words in particular because a list in an FR experiment can only comprise a limited number of words, so the distributional discrepancies would be even greater.

In other words, one *cannot* construct an experimental list of words the WF distribution of which would not be at odds with the WF distribution of the words in the participant’s native language. There are important consequences to this situation. One such consequence is that one cannot experimentally evidence the effect of such WF distribution discrepancy other than by measuring it. More importantly, one cannot control its effect (in the sense of partialling out its undue influence) but by measuring it and including it as a variable in all the statistical models that test for the putative effect of *other* variables of interest (e.g., WF, age of acquisition, etc.). Finally, if WF exerts its influence on FR performance through the WF distribution discrepancy we discussed, no (additional) WF effect should be found once one controls for the variable that measures the WF distribution discrepancy (i.e., with this latter variable included in the statistical model).

### Priors, expectations, surprisal and Bayesian surprise

Our view builds heavily on predictive coding theory (e.g., Rao and Ballard, 1999; Friston, 2003; Bubic et al., 2010; Huang and Rao, 2011; Clark, 2013). Initially developed based on findings in the field of perception (e.g., Srinivasan et al., 1982), predictive coding theory consists of the idea the brain is “using top-down connections to try to generate, using high-level knowledge, a kind of “‘virtual version”’ of the sensory data via a deep multilevel cascade… [with] the top-down flow as attempting to predict and fully “‘explain away”’ the driving sensory signal, leaving only any residual “‘prediction errors”’ to propagate information forward” (Clark, 2013). Later on, predictive coding theory was extended to action and motor control (e.g., Brown et al., 2011; see also free energy theory: Friston and Stephan, 2007; Friston et al., 2009; Friston, 2010) and lately to cognition in general (e.g., Lupyan and Clark, 2015; Spratling, 2016). For instance, Lupyan and Clark (2015) conclude that “predictive processing [i.e., processing in a model that implements hierarchical predictive coding] thus provides a plausible mechanism for many of the reported effects of language on perception, thought, and action.” We would like to add free recall to that. While we do not endorse all the assumptions of predictive coding, we do agree with the general idea the brain continuously predicts what is coming next and possesses the neural circuitry necessary to sense any significant divergence between its predictions and what actually occurs, and then adjusts itself (i.e., learns, memorizes) to the differences found between what was predicted and what occurred. In other words, its learning depends positively on the unexpected: the more unexpected an occurrence, the more learning/memorization is induced.

What bears special relevance to our proposal is the fact that “prediction error, … the divergence from the expected signal,… reports the “‘surprise”’ induced by a mismatch between the sensory signals encountered and those predicted” (Clark, 2013). Clark (2013) very appropriately stresses that “[m]ore formally – and to distinguish it from surprise in the normal, experientially loaded sense – this is known as *surprisal* (Tribus, 1961)”. This is a crucial point since Tribus’ *surprisal* has nothing to do with the notion of cognitive surprise as it has been construed since the late 1960s. The latter, *cognitive* surprise is best described in Kamin’s (1969) words: “[…] perhaps […] it is necessary that the US [unconditioned stimulus] instigate some mental work on the part of the animal. This mental work will occur only if the US is unpredicted, *if it* in some sense *surprises the animal*” (Kamin, 1969, p. 293, our emphasis). Distinguishing surprisal from “cognitive” surprise is necessary in order to avoid the pitfall of implying that participants have to be able to report experiencing surprise for there to be an effect. Indeed, while a rat that receives the first electric shock of its life may be surprised in the phenomenological sense (i.e., it may experience surprise in addition to its brain experiencing surprisal), there is no need to suppose that learning can be modulated only by something the learner (e.g., a human participant) is conscious of (i.e., surprise).

More recently Itti and Baldi (Itti and Baldi, 2009; Baldi and Itti, 2010) introduced the concept of *Bayesian surprise*, which seems virtually identical to that of Tribus’ *surprisal* but has the advantage of bringing the Bayesian framework into play, along with a mathematical definition and the possibility of making numeric predictions: “The amount of information contained in a piece of data can be measured by the effect this data has on its observer. Fundamentally, this effect is to transform the observer’s prior beliefs into posterior beliefs, according to Bayes theorem.” (Baldi and Itti, 2010). This relates well to predictive coding: “Predictive coding […] still depends upon known priors.” (Friston, 2003). The question that must be answered then is what the known priors are and where they come from in the case of (memorizing and recalling) words.

To answer this question, we begin by taking a broadly ecological approach to the issue (Gibson, 1979; Turvey et al., 1981; Agre, 1997; Chemero, 2009), according to which one must carefully examine environmental and informational resources available to the participant before positing any specialized cognitive processes. Moreover, according to this approach we adopt, informational and environmental resources are often higher-order, relational properties. This means we assume the neural structures that enable FR are determined by experience with a language but do not explicitly represent it, as a model or list, or searchable structure. Likewise, we do not assume the information on the frequency of the to-be-memorized words is available to the participants in an experiment, or that the statistical structure of the language (e.g., WF, but not only) is plausibly explicitly represented by the cognitive machinery of participants — so that participants’ cognitive machinery could make use of the frequency of the to-be-memorized words and yield the WF effects; in this sense, WF, as manipulated experimentally, is not *directly* responsible for the FR performance.

What is available to a participant in the FR task is a recently presented list of words, a cognitive system that has been shaped by their native language, and relations between these. Over developmental time the statistical structure of the language being learned and used shapes the neural structures in the same way that weather patterns and flowing water affects the landscape. The number of encounters with a word (operationalized by its WF) and other factors (e.g., what other words co-occurred, on what occasions, etc.) is responsible for the particular pattern of these neural structures. WF in the environment of the past linguistic experience is thus a *distal cause* with respect to FR performance, as it is (one of) the cause(s) that has been shaping the cognitive system over a long period of time and made it what it is at the time of the experiment (and its FR part). FR performance is then the end result of relational properties that preexist the experimental situation and of the way the words from the experimental list interact with those.^2^

Our account differs from extant models in that it does not posit specialized cognitive processes to account for FR. At its worst, positing a specialized cognitive process can seem overly *ad hoc* and, hence, unexplanatory. A single account of the relationship to FR that makes sense of both the positive and negative WF relations without positing multiple processes should be preferable. To achieve that, our strategy was to find the higher-order property of the environment the participants in the experiments are responding to. Only after we know exactly what information participants are using in order to recall words does it make sense to speculate about the cognitive and neural mechanisms that enable the recall (Gibson, 1979; Turvey et al., 1981; Agre, 1997; Chemero, 2009). In particular, we consider that the participants in FR experiments are not responding just to the to-be-recalled words of an experimental list, but to a particular relationship between the to-be-recalled words and the corpus of the language as a whole (with the latter having shaped participants’ cognitive system in a particular way), a relationship that we will capture through a proxy variable we call surprisal proxy (hereafter, SP).

Thus, the known priors, in the theoretical framework we propose, are provided by the particular neural structures of a participant that were previously shaped and determined by the statistical properties of the words in the participant’s native language. That a neural signal is triggered when something about the actual stimulus is at odds with the brain’s expectations has been convincingly argued for in neuroscience (e.g., Steinberg et al., 2013), so we contend that Bayesian surprise^3^, a neural signal, is triggered in a participant’s brain when some word statistical properties are at odds with brain’s expectations, given brain’s priors. Thus it is Bayesian surprise that makes some words from a word list more memorable and more prone to being recalled as compared to others. As mentioned before, Bayesian surprise is the end result of an interaction between a particular word in a given position in a list comprising those words in particular (and not comprising others), on one hand, and a brain the linguistic priors of which are what they are as a result of that participant being competently using their native language for many years prior to the experimental situation.

### Surprisal proxy, an experimental index of Bayesian surprise, and its interpretation

Because we have no direct means of measuring Bayesian surprise, we constructed an index of Bayesian surprise that we called surprisal proxy (SP) in order to test our view of FR performance, starting from the distributional properties of the words in the language and the to-be-recalled words in a list. SP is only intended as a variable that can be used to experimentally test our proposed account, and one should refrain from reifying SP into a concept or confounding it with Tribus’ notion of surprisal. This is the reason why we have not called SP Bayesian surprise: Bayesian surprise is the concept at work in our theoretical explanation, while SP is just a handy experimental proxy for it.

Before giving details on how the SP index is computed, some clarifications are in order. Firstly, we do not contend that FR performance is based on the computation of SP in the brain. Crucially, we assume no computation --- of the kind we will detail below when constructing our SP variable --- is carried out implicitly or explicitly by the participants. Secondly, the SP value for a to-be-remembered word on a list is not a transformation of that word’s frequency value. This is to say, that while for a given WF database, the WF value associated with a word is a fixed number^4^, the very same word will have SP values that will be low or high depending on which other words are in (and which are absent from) that list, and also on its order of occurrence with respect to the other words of the list. This latter point is made more manifest in the following presentation of the computation of SP.

Because WF is a discrete variable, computation of SP is carried out by intervals. Any given WF interval will include a certain number of words in the language, and potentially one or more words of the word list. The larger the considered WF interval, the larger will be the number of words in the language that are comprised within it. If no discrepancy exists between list and language in the distribution of WF, a large WF interval will also include a proportionally large number of words from an experimental word list. Another parameter to consider in the computation of SP is the particular form of the WF distribution (see Figure 1), which makes it such that for two WF intervals of equal widths, a (much) higher number of words in the language will fall into a WF interval situated in the low WF values — compare the leftmost and the second leftmost bins, or, more extremely, the leftmost and the rightmost bins in Figure 1. As there are far fewer words in the experimental list as compared to the words in the language, the words in the experimental list are used to define the WF intervals. The general idea of the computation of SP is the following (numeric examples follow):

(i) a given WF interval considered generally comprises a single experimental word (the exception being if there are two or more words of the exact same WF in the experimental word list); given the number of words in the word list, we can compute the percentage of the words from the list that are comprised in that WF interval, PctList (PctList is then the number of list words that fall within the interval divided by the total number of words in the list);

(ii) we determine how many words fall within that same WF interval in the language, and given the total number of words in the language, we can compute the percentage of the words from the language that are comprised in that WF interval, PctLanguage. Because the total number of words in the language may be very high in comparison to the number of words in the language that fall in the considered WF interval, PctLanguage is generally very low so a further step consists in taking the square root of PctLanguage, sqrt(PctLanguage);

(iii) the width of the interval, WInt is taken into account;

(iv) we compute the ratio of sqrt(PctLanguage) to PctList, then we divide by WInt; the log of the result is SP. In other words, for one word (or many words of the exact same WF) of the experimental list we have


$$S\,P=l\,o\,g\,\left(\frac{\frac{\sqrt{\left(\mathrm{PctLanguage}\right)}}{\text{PctList}}}{\text{WInt}}\right)$$

$$=l\,o\,g\,\left(\frac{\sqrt{\left(\mathrm{PctLanguage}\right)}\times \mathrm{Wint}}{\text{PctList}}\right)$$

There are however a number of additional important details to be taken into account in the computation of SP. The first step in computing SP is defining the distribution of words in the language that we take as reference. Considering an experiment in which the words in the experimental lists are French nouns in singular form, we would take as a reference all nouns in French in the singular form (hereafter called only ‘words’, for simplicity’s sake) of all frequencies^5^. There are 24,530 French nouns in the singular form in the database we used, LEXIQUE (New et al., 2001, 2004), the reference database for frequency of use of words in adults in French. Next, one must ensure the distribution of our reference population of words follows a Zipfian distribution, that is, the relation between the ranks of the words and their frequency of use follows a decaying exponential law. This was indeed the case, as a decaying exponential model explains 99.84% of the variance in the data (*p* < 0.0001).

Importantly, the computation of SP takes into account the words so far presented to a participant in the experiment, which means a word’s SP value depends on that word’s position in the list. Let us suppose the first six words of a 30-word list that are presented to a participant are, in their order of presentation, *plongeur* (diver), *cercle* (circle), *brouette* (wheelbarrow), *esquimau* (eskimo), *poireau* (leek) and *oie* (goose), and that their respective word frequencies are 1.69, 42.43, 5.14, 0.88, 0.88, and 5.2 (occurrences per million words, from LEXIQUE). The word *plongeur* adds one word of its frequency to the experimental list (this general formulation is introduced to take into account the case of two or more words that follow immediately each other and have the exact same frequency), and the interval it is associated with is [0.07^6^; 1.69], that is, an interval of width 1.62. In this interval, there are 12,513 words in French, and there are a total of 24,530 French words in this example. The SP value for *plongeur* as the first word in the list is thus log{[sqrt(12,513/24,530)*1.62]/(1/30)}, that is about 1.54. The word *cercle* is associated with the interval [1.69; 42.43] because the inferior bound is given by the word with the highest frequency i) that has already been presented and ii) the frequency of which does not exceed that of the word at hand — here by the frequency of the first word, *plongeur*. In this interval, there are 6,204 words, and the width of this interval is 40.74. The SP value for *cercle* as the second word in the list is thus of log{[sqrt(6,204/24,530)*40.74]/(1/30)}, that is about 2.79. The SP value for *brouette* as the third word in the list is about 1.58 (there is nothing noteworthy about its computation). The words *esquimau* and *poireau* add to the experimental list two words of the exact same frequency and the interval they are associated with is [0.07; 0.88], an interval of width .81 and containing 10,037 words. The SP value for both *esquimau* and *poireau* as the fourth and fifth words in the list is thus of log{[sqrt(10,037/24,530)*0.81]/(2/30)}, which is about 0.89. Finally, the word *oie* adds one word of its frequency to the experimental list and the interval it is associated with is [5.14; 5.2], an interval containing 29 words, and the width of this interval is 0.06. The SP value for *oie* as the sixth word in the list is thus of log{[sqrt(29/24,530)*0.06]/(1/30)}, which is about −1.21.

The interpretation of an SP value is quite straightforward. One may very loosely think of the numerator of SP, sqrt(PctLanguage), as “what is expected” and of the denominator, PctList, as what is observed in the experimental list. If the interval width and/or the number of words in the language that fall within an interval is/are low (cf. word *oie*), little is expected, and finding that one word of 30 the list comprises falls in that interval should not generate any Bayesian surprise because, if we may say, “nothing is lacking with respect to what was expected” (SP is of about −1.21). On the contrary, the word *cercle* falls into a very large interval that comprises many words in the language. *This makes manifest that one or more words of a lower WF were expected and were not present in the list*. When the word *cercle* is presented, it entails the absence of all those other words of lower WF from the experimental list, which generates Bayesian surprise. That Bayesian surprise is associated with the word *cercle* (SP is about 2.79) — that word is present when Bayesian surprise occurs, and there is nothing else to associate Bayesian surprise with at that time. It is crucial to understand that there is nothing surprising about the word *cercle*. What *is* surprising to the cognitive system is that something else was expected (before a word of such a “high” WF) and that expectation is contradicted by the very presence of the word *cercle*: whatever was expected did not occur, the word *cercle* was presented instead. Based only on their SP values, among the six words in our example, the one that is expected to have the highest (lowest) recall probability is *cercle* (*oie*), with an SP of about 2.79 (−1.21).

## Experiments

The approach we opted for in the experiments described below is motivated by the following. Firstly, as previously discussed, one cannot compare two conditions, one with a list of words that conforms and the other list of words that do not conform to the Zipfian distribution in the language, and observe when a WF effect on FR performance is obtained. One is thus left with the option of controlling the effect of these discrepancies at a statistical level. We introduced and defined the SP variable for this reason, as a proxy for the Bayesian surprise, which we suppose is derived by the brain from the distributional discrepancies. When testing for the influence of a variable (e.g., WF) on FR performance, we will do so with SP in the model.

The possible outcomes are thus the following. If participants’ cognitive systems are *not* sensitive to the discrepancies that exist between the WF distributions in the language and in the experimental lists, SP will not be a predictor of FR performance. This is a perfectly valid prediction despite being a null hypothesis prediction because we will carry out all statistical analyses in a Bayesian framework that allows for validating the null hypothesis if the model derived from it fits the data best. In this case, if WF is indeed a genuine predictor of FR performance, if it exerts its influence in a way different from what we propose here, the effect of WF should manifest itself.

On the other hand, if SP explains FR performance, that is, there is no WF effect above and beyond that of SP, then one must conclude that the manipulation of WF does not have a direct effect on FR performance. In this case, WF manipulations rather affect FR performance by creating statistical discrepancies that are detected by the brain, which by the mechanism of Bayesian surprise makes some words more memorable.

These predictions are tested in two classic FR experiments where participants are first asked to memorize a list of words that are presented to them one at a time and then asked to recall the memorized words in whatever order these come to their mind. For both these experiments, we chose a reasonably high number of words per list in such a way as to make sure there is enough variability for each and all different descriptors/predictors (i.e., WF, age of acquisition, etc.; see Experiment 1), in order to be able to test for the influence of each of these descriptors on FR performance in a within-subjects design.

### Experiment 1

Experiment 1 was a proof of concept and as such suffers from some shortcomings. Among these, a small number of participants, probably too extensive a backward counting task between the memorization phase and the FR phase, and a programming error that led to the presentation of the same first four words of a list always in the same order. We decided to include it because it yielded some remarkable results.

#### Method

##### Participants

A total of fourteen participants (4 men) aged 19-25 years (mean = 20.65; SD = 1.63), all second-year psychology students at the University Rennes 2 (Rennes, France), participated in the experiment for course credit. They were all native speakers of French and were randomly assigned to one of the two experimental groups (see next section) so they memorized and recalled a single list of words.

##### Stimuli and apparatus

The words used as stimuli (cf. Appendix A) are 72 concrete and quite common French words that are names of objects presented as line drawings in Snodgrass and Vanderwart’s (1980) set and are also found in Alario and Ferrand (1999). The words are basic enough to be deemed suitable as experimental material in patients with anomia (e.g., McCarthy and Kartsounis, 2000) and as therapeutic material in patients with post-stroke aphasia (Macoir et al., 2017). The particular words retained here were chosen so as to maximize the variability along the WF dimension and also because a full set of descriptors exists for each of them — in French, there are only a few hundred words for which all the descriptors mentioned below are available. Two equivalent lists of 36 words were constructed (L1 and L2, cf. Appendix A) and each participant was presented randomly with one of the lists (seven participants saw L1).

The word descriptors were the following. Number of phonemes (PhN) and printed frequency (WFA) come from LEXIQUE (New et al., 2001, 2004). The frequency of use of words in books for French children (WFC) is taken from the MANULEX database (Lété et al., 2004). Age of acquisition (AoA), the age at which a speaker first knows consistently the meaning of a word, is taken from Chalard et al. (2003). Conceptual familiarity (CFam), the familiarity of the concept a word refers to, is based on Bonin et al. (2003). We also included as predictor animacy (Anim), a binary variable that opposes animate to inanimate things, because animacy was found to influence performance in a naming task (see Howard et al., 1995), possibly because it is information that is available before name phonology for the object to be named (e.g., van Turennout et al., 1997). For SP, our last predictor, note that a given word does not have a fixed SP value, its SP value depends on that word’s position in the list, the words the list contains, which of those were already presented, and the words that are not present in the list.

As no correlation was different from one experimental list to the other (all *p* > .05), pairwise correlations between all numeric descriptors are presented in Table 1 for both experimental lists combined^7^ (point-biserial correlation was used to correlate the dichotomous animacy variable with the other numeric variables).

**TABLE 1:** Pairwise correlations between the word descriptors considered in Experiment 1.

	WFA	log_10_WFA	SP^ (1)^	AoA	log_10_AoA	CFam	log_10_CFam	WFC	log_10_WFC	PhN
log_10_WFA	0.803***									
SP^ (1)^	0.496***	0.492***								
AoA	−0.410**	−0.514***	−0.314***							
log_10_AoA	−0.437***	−0.512***	−0.301***	0.988***						
CFam	0.086	0.086	0.070	−0.253*	−0.243					
log_10_CFam	0.093	0.074	0.065	−0.251	−0.239	0.985***				
WFC	0.846***	0.675***	0.426***	−0.434***	−0.465***	0.060	0.060			
log_10_WFC	0.565***	0.657***	0.459***	−0.653***	−0.625***	0.092	0.098	0.649***		
PhN	−0.214	−0.375**	−0.123*	0.335**	0.345**	0.065	0.077	−0.218	−0.218	
Anim	0.074	−0.033	0.119*	−0.209	−0.197	−0.200	−0.215	0.217	0.255*	0.086

*: p < 0.05; **: p < 0.01; ***: p < 0.001. (1): see footnote 7. Numbers on last row are point-biserial correlations. See main text for details.

The task was driven by E-prime 2.0 (PST Inc., PA, United States), on an IBM-compatible computer running Windows and using a 3:4 ratio 17” screen. Stimuli were presented on a black background in white lowercase 24-sized bold Courier New font characters. They were displayed centered both horizontally and vertically on the screen. Participants sat in front of the screen at an approximate viewing distance of 60 cm.

##### Design and procedure

WF and other word descriptors vary within-subject. Each participant was tested individually. Participants were welcomed and received the instructions on the computer screen. They were instructed to memorize all the words that were going to be presented to them and were told they would have to recall those words but that they would not be required to recall them in the order they were presented to them.

Each stimulus was presented once, for 3,000 ms, and was followed by a black screen for 1,500 ms. The first four words of the list^8^ and the other 32 words (always presented in a random order that differed from one participant to another) were presented without interruption so that nothing distinguished the former ones from the later ones. After the last word of the list was presented, a message indicated to the participant that the presentation of the to-be-memorized words was over and the participant was then instructed to count backward in threes from 300 (i.e., 300, 297, 294, etc.). The participant carried out this task for 120 s, then was handed a sheet of paper and a pencil and asked to write down all the words they could remember. They had 5 min to complete the FR task.

#### Results

In order to avoid confounding an effect of the order of presentation with that of a word descriptor, the four low WF words that were presented always in the same order at the beginning of the list to all participants (see the previous section) were excluded from the analyses. Out of the thirty-two remaining words, the number of free recalled words varied between four and eighteen (mean = 8.79, SD = 3.75).

All analyses that follow are carried out within a Bayesian model comparison approach that consists in building all models, comparing them, and retaining the model that shows the best fit to the data. Model fit to data is evaluated through the Bayesian Information Criterion (BIC: Schwarz, 1978), with the lowest BIC value reflecting the most probable model. All models are mixed-effect logistic regression models with a logit link on the binomial dependent variable (one that codes for whether a word was recalled or not, hereafter Resp), a random subject effect that allows taking into account the variations in mean performance between participants and possibly one or more other fixed-effect independent variables. Data analysis and interpretation were carried out with R (R Core Team, 2014) using the R2STATS GUI (Noël, 2014) based on the lme4 library (Bates et al., 2015).

The null model is formally written as:


(1) $$l\,n\,\left[\frac{\phi_{i\,j}}{\left(1-\phi_{i\,j}\right)}\right]=\beta_{0\,j}=\gamma_{00}+u_{0\,j},$$

with ϕ_*ij*_ = P(Resp_*ij*_ = 1| β_*j*_) and u_0j_∼N(0, ψ)

and an augmented model that includes a word descriptor (e. g., WF in adults, WFA) as a fixed-effect predictor is formally written as:


(2) $$l\,n\,\left[\frac{\phi_{i\,j}}{\left(1-\phi_{i\,j}\right)}\right]=\beta_{0\,j}+\beta_{1\,j}\,W\,F\,A_{i\,j}=\gamma_{00}+\gamma_{10}\,W\,F\,A_{i\,j}+u_{0\,j},$$

withϕ_*ij*_ = P(Resp_*ij*_ = 1| β_*j*_) and u_0j_∼N(0, ψ)

If we note the participant variable as Subj and we keep in mind that all models are mixed-effect logistic regression models with a logit link on the binomial dependent variable Resp, the same two models can be written more informally using the notation introduced by Bates et al. (2015) as Resp∼(1| Subj) (the null model formally defined in (1)), and, respectively, Resp∼(1| Subj)+WFA (the augmented model formally defined in (2)). For simplicity’s sake, we will use the later model notation throughout. Also for simplicity’s sake, we do not present here the more complex random slopes models we built (i.e., models where the values of the dependent variable are considered to vary not only as a function of the predictor to be tested while keeping the same slope for all participant, but also allows the supplementary degree of freedom that the slopes be different between the participants), because the results were the same and those more complex models did not fit the data better.

We first checked whether there was a list effect. In order to do that, we defined a model similar to that defined in (2), but including the List (L1 vs. L2) as a predictor (instead of WFC), that is, Resp∼(1| Subj)+List, and compared its BIC (BIC = 539.69) to that of the null model (BIC = 534.28) defined in (1), Resp∼(1| Subj). As the BIC for the null model is lower than that of the augmented model including List (i.e., the null model is the better of the two models), we can conclude that the performance does not depend on the list. Thus, all subsequent analyses are carried out on all data, irrespective of the list.

In what follows, the details of the models mentioned are given in Table 2. The next analysis concerned the existence of order effects. We considered a linear effect of word order in the list (M2) and a quadratic one (M3) but both models were less good than the null model (M0). The lack of primacy and recency effects is somewhat surprising, but less so if one considers the first four words that were systematically presented in the same order were discarded from the analyses (which may have led to the absence of a primacy effect) and also that the word presentation was followed by a 2-min backward counting task (which may have decreased enough the recency effect so as to mask it).

**TABLE 2:** Models and model fit (BIC) to the data of Experiment 1.

Model #	Model details	BIC
M0	Resp∼(1| Subj)	534.28
M1	Resp∼(1| Subj)+List	539.69
M2	Resp∼(1| Subj)+Order	536.28
M3	Resp∼(1| Subj)+Order+(Order)^2^	541.84
M4	Resp∼(1| Subj)+WFA	537.76
M5	Resp∼(1| Subj)+log_10_WFA	537.42
M6	Resp∼(1| Subj)+AoA	537.36
M7	Resp∼(1| Subj)+log_10_AoA	537.01
M8	Resp∼(1| Subj)+PhN	539.17
M9	Resp∼(1| Subj)+CFam	537.62
M10	Resp∼(1| Subj)+log_10_CFam	538.74
M11	Resp∼(1| Subj)+CFam+(CFam)^2^	539.83
M12	Resp∼(1| Subj)+WFC	537.72
M13	Resp∼(1| Subj)+log_10_WFC	537.46
M14	Resp∼(1| Subj)+WFC+(WFC)^2^	543.37
M15	Resp∼(1| Subj)+log_10_WFC+(logWFC)^2^	541.91
M16	Resp∼(1| Subj)+SP	529.53
M17	Resp∼(1| Subj)+Anim	532.27
M18	Resp∼(1| Subj)+SP+Anim+SP:Anim	531.68
M19	Resp∼(1| Subj)+SP+Anim	529.29

The “:” sign appearing in model M18 means that model also includes as a predictor the interaction term SP by Anim.

Having established there were no order effects, we turn now to the one-by-one analysis of all the word descriptors. These were considered under their raw form or as log-transformations of these, and models considered their linear effect. In addition, when graphically the relationship between a predictor and the dependent variable seemed to have a quadratic form, a model considering a quadratic effect was also defined and tested. The models considering as a predictor adult WF, a linear version (M4), or its log-transformation (M5) are both less good that the null model (M0), which means that WF cannot explain the data. The same is true for words’ age of acquisition (models M6 and M7), word length (number of phonemes; model M8), conceptual familiarity (models M9, M10, and M11), and child WF (models M12, M13, M14, and M15).

Considering as a predictor SP yields a model (M16) that is better than the null model (M0). The other word descriptor that seems to explain the performance is animacy (cf. M17), with a better recall for animate than for inanimate things (39.56% vs. 24.37% mean percent recall). A model that includes both predictors (i.e., SP and Anim) under an interactive effect assumption (M18) does not account better for the data than the model that supposes only an effect of SP (i.e., M16). The same is true for a model that includes both predictors under an additive effect assumption (M19)^9^.

**FIGURE 3:**
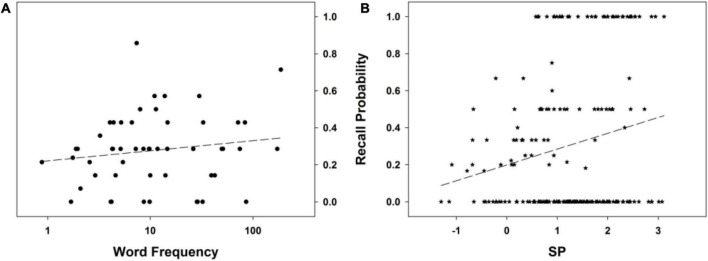
Free recall probability as a function of **(A)** word frequency in adults (log scale), and **(B)** SP. The dashed line stands for the mean probability of the recall curve. See text for details.

To summarize, no word descriptor among those considered — including WF — other than SP explains the FR performance in this experiment. The best model involving adult WF as a predictor (although, again, not better than the null model), Resp∼(1| Subj)+log_10_WFA, has a poor fit to the data (this conclusion is confirmed by the inspection of Figure 3A). On the other hand, SP exhibits a clear relationship to FR performance (see Figure 3B), one that is monotonic (the model, Resp∼(1| Subj)+SP, tests for a linear effect of SP). This supports our idea that the higher the Bayesian surprise associated with a word, the higher its probability of subsequent recall. The next experiment will further put to test this view.

### Experiment 2

No WF effect was evidenced in Experiment 1 (while SP had a significant effect on FR). This may have been a consequence of the low number of participants in that experiment (i.e., by lack of statistical power). Accordingly, significantly more participants took part in Experiment 2. However, Experiment 2 is not just a replication of Experiment 1 with more participants. Unlike Experiment 1, Experiment 2 includes a dummy task, presented to the participants before the memorization phase of the FR experiment, that introduces a manipulation aimed at drawing participants’ attention to WF. This manipulation aims at favoring the apparition of a WF effect on FR performance.

#### Method

##### Participants

A total of forty-five participants (7 men), aged 19-27 years (mean = 21.23; SD = 1.89), all second-year psychology students at the University Rennes 2 (Rennes, France), participated in the experiment for course credit. They were all native speakers of French and were assigned randomly to one of the two experimental groups (see next section). No participant took part in Experiment 1.

##### Stimuli and apparatus

Two equivalent lists of 34 words, L1’ and L2’ are used (twenty-four participants saw L1’). The words are those of lists L1 and L2 from Experiment 1 except for two stimuli per list, which were discarded. The discarded words are *haltère* (dumbbell, WFA = 0.07) and *astronaute* (astronaut, WFA = 0.14) from L1, and *lance* (spear, WFA = 0.14) and *trombone* (trombone, WFA = 0.54) from L2. This change with respect to Experiment 1 was introduced because it was suggested to us that there were too many words in the list to be memorized and also too many low-frequency words, with some of them having a particularly low WF. Each participant was presented randomly with one of the lists. The word descriptors considered are the same as in Experiment 1, as is the apparatus and stimuli presentation details. As in Experiment 1, no correlation was different from one experimental list to the other (all *p* > 0.05), so pairwise correlations between all numeric descriptors (SP included) are presented in Table 3 for both experimental lists combined.

**TABLE 3:** Pairwise correlations between the word descriptors considered in Experiment 2.

	WFA	log_10_WFA	SP^ (1)^	AoA	log_10_AoA	CFam	log_10_CFam	WFC	log_10_WFC	PhN
log_10_WFA	0.776***									
SP^ (1)^	0.529***	0.501***								
AoA	−0.417***	−0.614***	−0.285***							
log_10_AoA	−0.450***	−0.597***	−0.291***	0.985***						
CFam	0.126	0.219	0.034	−0.378**	−0.350**					
log_10_CFam	0.136	0.222	0.027	−0.393***	−0.359**	0.986***				
WFC	0.848***	0.657***	0.448***	−0.429***	−0.469***	0.096	0.100			
log_10_WFC	0.531***	0.686***	0.352***	−0.661***	−0.639***	0.211	0.228	0.604***		
PhN	−0.235	−0.422***	−0.179***	0.389**	0.393***	−0.012	−0.007	−0.233	−0.233	
Anim	0.067	−0.044	0.094***	−0.180	−0.175	−0.176	−0.180	0.202	0.204	0.107

*: p < 0.05; **: p < 0.01; ***: p < 0.001. (1): see footnote 7. Numbers on last row are point-biserial correlations. See main text for details.

##### Design and procedure

The classic FR experiment (i.e., memorization of a list of words, filled delay, FR of the memorized words) occurred after a short dummy task intended to favor the participant’s processing of the WF dimension of words. This task was presented to the participants as a computerized vocabulary test. It was a go/no-go task, with 24 words (presented one at a time) that were either from one of the two lists (e.g., 8 words of L1’ if list L2’ was the list of words that participants had to memorize later) or extremely rare words (e.g., *pyrargyrite*, *ophiolite*, *ptérostygma*; see Appendix B) and participants’ task was to press a key as fast as possible each time they saw a word the meaning of which they didn’t know. Each word was presented for a maximum of 3,000 ms or until a key was pressed. Upon completion of the go/no-go task, the participants received the instructions for the FR task, which were the same as in Experiment 1. Each to-be-memorized word was presented once, for 3,000 ms (24 participants, of whom 12 saw L1’) or 3,500 ms (21 participants, of whom 12 saw L1’) and was followed by a black screen for 1,500 ms. Words’ order of presentation was randomized for each participant. WF and other word descriptors vary within-subject. Each participant was tested individually. After the last word of the list was presented, a message indicated to the participants that the presentation of the to-be-memorized words was over and that they had 1 min to rehearse the words. After 1 min, participants were instructed to count backward in threes from 300. The participants carried out this task for 60 s, then were handed a sheet of paper and a pencil and asked to write down all the words they could remember. They had 3.5 min to complete the FR task.

#### Results

The number of free recalled words varied between eight and 27 (mean = 14.31, SD = 4.39) out of 34. It is noteworthy that in this experiment recall performance is better than in the previous experiment, with a mean recall performance of 38.74% (27.46% in Experiment 1), BIC_*different*_ = −59.81, BIC_*same*_ = −56.50 (BF_10_ = 5.25). The following data analysis and interpretation were carried out within the framework defined in the analogous section of Experiment 1. As for the analyses of Experiment 1, the more complex random slopes models we built are not presented here because they yielded the same results and did not fit the data better.

The details of the models mentioned hereafter are given in Table 4. We first checked whether there was a list (L1’ vs. L2’) effect or/and a word presentation time (3,000 vs. 3,500 ms) effect, or an interactive effect of these. As can be seen from the comparison of models M0 to M4, the two lists are equivalent and FR performance does not differ between the two presentation times that were used. Indeed, the model includes List as a predictor (M1), the model including presentation time (PTime) as a predictor (M2), the additive model including List and presentation time as predictors (M3), and the interactive model including List and presentation time as predictors (M4) all have a less good fit to the data than the null model (M0). Therefore, all subsequent analyses are carried out on all data, irrespective of the list and word presentation time.

**TABLE 4:** Models and model fit (BIC) to the data of Experiment 2.

Model #	Model details	BIC
M0	Resp∼(1| Subj)	2,076.43
M1	Resp∼(1| Subj)+List	2,082.42
M2	Resp∼(1| Subj)+PTime	2,079.42
M3	Resp∼(1| Subj)+List+PTime	2,085.62
M4	Resp∼(1| Subj)+List+PTime+List:PTime	2,092.94
M5	Resp∼(1| Subj)+Order	2,082.24
M6	Resp∼(1| Subj)+Order+(Order^2^)	2,025.63
M7	Resp∼(1| Subj)+Order+(Order^2^)+Anim	2,031.00
M8	Resp∼(1| Subj)+Order+(Order^2^)+CFam	2,031.96
M9	Resp∼(1| Subj)+Order+(Order^2^)+log_10_CFam	2,032.49
M10	Resp∼(1| Subj)+Order+(Order^2^)+PhN	2,029.47
M11	Resp∼(1| Subj)+Order+(Order^2^)+log_10_PhN	2,026.86
M12	Resp∼(1| Subj)+Order+(Order^2^)+WFA	2,022.57
M13	Resp∼(1| Subj)+Order+(Order^2^)+log_10_WFA	2,020.40
M14	Resp∼(1| Subj)+Order+(Order^2^)+SP	2,012.87
M15	Resp∼(1| Subj)+Order+(Order^2^)+AoA	2,025.92
M16	Resp∼(1| Subj)+Order+(Order^2^)+log_10_AoA	2,022.63
M17	Resp∼(1| Subj)+Order+(Order^2^)+AoA+(AoA)^2^	2,019.80
M18	Resp∼(1| Subj)+Order+(Order^2^)+WFC	2,025.61
M19	Resp∼(1| Subj)+Order+(Order^2^)+log_10_WFC	2,030.63
M20	Resp∼(1| Subj)+Order+(Order^2^) +WFC+(WFC)^2^	2,032.15
M21	Resp∼(1| Subj)+Order+(Order^2^) +log_10_WFC +(log_10_WFC)^2^	2,022.61
M22	Resp∼(1| Subj)+Order+(Order)^2^ +log_10_WFA+SP+AoA +(AoA)^2^+log_10_WFC+(log_10_WFC)^2^	2,032.22
M23	Resp∼(1| Subj)+Order+(Order)^2^ +log_10_WFA+SP+AoA +(AoA)^2^	2,020.70
M24	Resp∼(1| Subj)+Order+(Order)^2^ +log_10_WFA+SP+log_10_WFC +(log_10_WFC)^2^	2,027.39
M25	Resp∼(1| Subj)+Order+(Order)^2^ +log_10_WFA+AoA+(AoA)^2^*CPSTABLEENTER* + log_10_WFC+(log_10_WFC)^2^	2,026.42
M26	Resp∼(1| Subj)+Order+(Order)^2^ +SP+AoA+(AoA)^2^*CPSTABLEENTER* + log_10_WFC+(log_10_WFC)^2^	2,030.90
M27	Resp∼(1| Subj)+Order+(Order)^2^ +log_10_WFA+SP	2,018.86
M28	Resp∼(1| Subj)+Order+(Order)^2^ +log_10_WFA+AoA+(AoA)^2^	2,019.66
M29	Resp∼(1| Subj)+Order+(Order)^2^ +SP+AoA+(AoA)^2^	2,014.48

The “:” sign appearing in model M4 means that model also includes as a predictor the interaction term List by PTime.

The next analyses concern the existence of order effects. The fit of a linear order model (M5) is not better than that of the null model (M0). The model of a quadratic effect of word order in the list (M6) has a better fit to the data than the null model (M0). Indeed, substantial primacy and recency effects are present (though the latter was less marked). For this reason, the quadratic effect of the word order model (M6) will serve as a comparison point for the other models and all other variables will be tested in a model including a quadratic effect of word order.

We turn now to the one-by-one analysis of all the word descriptors. These were considered under their raw form or as log-transformations of these and models considered their linear effect. In addition, when graphically the relationship between a predictor and the dependent variable seemed to have a quadratic form, a model considering a quadratic effect was also defined and tested. The model including animacy as a predictor (M7) has a less good fit to the data than the reference model (M6), which means that animacy cannot explain the FR data. The same is true for conceptual familiarity (models M8 and M9) and for word length (number of phonemes; models M10 and M11).

**FIGURE 4:**
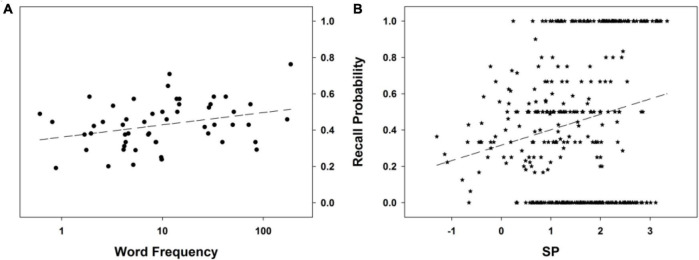
Free recall probability as a function of **(A)** log-transformed adult word frequency, and **(B)** SP. The dashed line stands for the mean probability of the recall curve. See text for details.

The models considering as a predictor adult WF, a linear version (M12) or its log-transformation (M13), are both better than the reference model, with the latter being the best of them (see Figure 4A), BF_10_ = 13.67 (”positive evidence”: Jeffreys, 1961; Raftery, 1995). The model considering an effect of SP (M14) fits the data better than the model of reference (M6) too. As in Experiment 1, the relationship between SP and FR is one such as the higher the Bayesian surprise, the higher the probability of subsequent recall of a word (see Figure 4B). For words’ age of acquisition and child WF, there are also models that fit better the data than the model of reference: a quadratic effect model of words’ age of acquisition (M17) fits the data better than models considering a linear effect (M15 and M16); the same is true for child WF, with the best model being M21 — the other models being M18, M19, and M20.

Log-transformed adult WF and SP are not the only word descriptors that explain (part of) FR performance when considered alone in a model (in addition to the quadratic order effect). The other candidates are i) a quadratic effect of the age of acquisition, and ii) a quadratic effect of the log-transformed child WF. In order to test which of these effects are genuine and which are not, we first created a model that includes them all and then pruned it, that is, took away one predictor at a time from that model. If the model’s fit is improved when a predictor is not in the model, that predictor does not explain a significant part of deviance. On the contrary, if the model’s fit deteriorates when a predictor is taken out of the model, that predictor does explain a significant part of deviance and should be kept in the model. Applying recurrently this reasoning is guaranteed to produce the best and most parsimonious model. Our initial model is thus M22. As its BIC value is higher than that of the reference model (M6), this is not the best model, so we test the other models that can be derived from it by taking away one predictor at a time. There are four such models, M23-M26. Of these four models, the first one (M23) has the best fit, and it not only improves over the initial model but also has a better fit than the reference model; starting from this model as the provisionally best model, we prune it and test the other models that can be derived from it by taking away one predictor at a time. There are now three such models, M27-M29. As one can see from Table 4, among these three models there is a model, M29, that is better than both the model with one more predictor (M23) and the other models with the same number of predictors (M27 and M28). It is noteworthy that model M29 is the one model among the three that does not include adult WF. In other words, leaving adult WF out of the model improves the model’s fit to the data.

If one takes away one of the two predictors of the latter and so far best model, M29, one ends up with two models that were already presented, M14 and M17. As the model that includes as predictors SP and the quadratic effect of a word’s age of acquisition (i.e., M29) yields a poorer fit than the simpler model that includes only SP as a predictor (M14), one can conclude that the only word descriptor that can be used on this data set to explain FR deviance is SP. Indeed, including in a model (in addition to the quadratic order effect), any other word descriptor in addition to SP leads to an increase in BIC value, which is synonymous with a poorer data fit. For instance, a model including both WF and SP, M27, has a poorer fit.

To summarize, no word descriptor (including WF) explains to a significant degree the FR performance in this experiment above and beyond SP, with the best model being M14, Resp∼(1| Subj)+Order+(Order)^2^+SP (BIC = 2012.87). Compared to the model of reference, M6, Resp∼(1| Subj)+Order+(Order)^2^ (BIC = 2025.63), there is “very strong [Bayesian] evidence” (Jeffreys, 1961; Raftery, 1995) that the augmented model, that is, the model that in addition includes SP, is the best model for the data (BF_10_ = 589; posterior probability = .9983). This supports our hypothesis that the higher the SP for a given word, the higher its probability of subsequent recall. Importantly, when SP is controlled for (i.e., is a predictor included in the model), WF does not influence FR performance. In conclusion, the results obtained show no WF effect above and beyond that of SP. This brings support to our claim that WF manipulations do not have a direct effect on FR performance, and that the effects that are then obtained are to be interpreted as rather arising from the distributional discrepancies that are created between the list words and the words in the language, and thanks to participants’ brains being sensitive to those discrepancies and generating Bayesian surprise, which then makes some words more memorable.

## General discussion

Attempts to relate word frequency (WF) to free recall (FR) performance lead to paradoxical findings, where depending upon the design of the experiment a range of conflicting results for the WF effect are observed. There are differences in the WF effect depending upon whether experimental word lists are “pure” (between-subjects design) of “mixed” (within-subjects design), and a parametric study that considered an analysis of FR performance across WFs revealed a non-monotonic ‘check mark’-shaped relationship with WF — one that, to our best knowledge, no extant theory can explain.

The beautiful minimalism of the free recall task and the relative simplicity of the explanations proposed so far in the literature to explain how WF affects participants’ performance in this very form of the FR task is what made us concentrate our endeavor on this task alone. Indeed, it lends itself well to the ecological psychology approach we adopt, and it was possible to identify the higher-order, relational properties that are the environmental and informational resources available to the participants. Our view is that participants in FR experiments are not responding just to the to-be-recalled words of an experimental list but to a particular relationship between the to-be-recalled words and the corpus of the language as a whole (with the latter having shaped participants’ cognitive system in a particular way). In particular, we consider that participants’ command of their native language implies implicit distributional knowledge of WF in the language, and when the distributional properties of words in an experimental list exhibit massive enough discrepancies with respect to those in the language, Bayesian surprise is triggered, which then marks some words and makes them more memorable. In order to test this view, we proposed a way to compute a proxy for Bayesian surprise, which we called surprisal proxy (SP).

SP is computed following the idea that, given the Zipfian distribution of WF, it would be surprising to have, in a mixed-WF list, a very high WF word presented as, say, the second word in the list if the first was an LF word because there are so many low and medium WF words in the language that could have been presented instead. In other words, there is nothing surprising about being that very high WF word in our example, what is surprising is that other words of lesser WF were expected to occur in place of it and did not. As the high WF word in our example is presented, the Bayesian surprise associated with the absence of a word of lesser WF in place of the high WF word is generated and marks the high WF word.

We have built on the idea that triggering a Bayesian surprise signal in the brain in this situation relies on “what is missing”. This seems to be very counterintuitive^10^, so we illustrate how this is the case with some numerical examples. When picking out a word at random (following the Zipfian distribution), it is approximately 6.624 times more probable to get a low or medium WF word than a word from the rightmost three bins from Figure 2A. One may object that very low WF words may not be known to most participants, which biases the computation of the odds ratio we computed — and possibly when excluding such unknown words, the odds ratio would be in favor of the highest WF words. This is however not the case. If we exclude all words that have a WF of less than 1 per million (and there are 18,294 such words out of the total of 30,451 nouns!), it is still approximately 6.47 times more probable to get a medium-low or medium WF word than a word from the three bins of highest frequencies. Exclude all words that have a WF of less than 20 per million (and there are 28,276 such words out of the total of 30,451 nouns), and it is still approximately 5.126 times more probable to get a medium WF word than a word from the three bins of highest frequencies. As counterintuitive as this may be, the number of low and medium WF words outweighs the massive occurrence of the (very) few (very) high WF words. It is thus not very probable to get a (very) high-frequency word when the random choice takes into account the Zipfian distribution of WF.

Our prediction was that the higher the SP value associated with a word, the higher its probability of free recall. If our reasoning that led to the introduction of SP as a variable is correct, then no WF effect will be found on FR performance above and beyond the effect of SP. If not, a genuine effect of WF will be found, be it accompanied by an effect of SP or, more probably, without any significant effect of SP. In any case, given its potential effect on FR, SP has to be a predictor in the statistical model that tests for the effect of WF on FR performance.

Experiment 1 has quite some shortcomings but we decided to include it for a reason that we discuss here. Firstly, as can be deduced from the FR performance, the task was quite hard, maybe because there were 36 words to memorize. The first four words of the lists used occurred always in the same order and thus had to be discarded, stripping the participants’ data of the primacy effect; the backward counting task the participants had to carry out after completion of the presentation of the list of words was quite extensive, which led to no recency effect being present. Most importantly, the experiment was underpowered, with only fourteen participants. This, in addition to the fact that a WF effect is not always found in a mixed list design, may well explain why a WF effect was not obtained; for these reasons, we refrain from commenting on the absence of a WF effect. What is interesting to note is that the expected effect of SP was found. Indeed, even though the experiment included only fourteen participants, it was found that SP has a significant effect on FR performance, with a positive relationship between the two variables.

Experiment 2 eliminated the shortcomings noted in Experiment 1. Crucially, Experiment 2 had enough power to yield an effect of WF in a model that did not include SP as a predictor — which is the way tests for an effect of WF have been carried out until now. However, when starting from a model that includes each and all predictors that seem to influence FR performance when alone in a model, and then pruning it to obtain the model that fits best the data, the model improved when WF was excluded. Importantly, if one carries out the same pruning technique without entertaining the possibility that SP plays a role (i.e., without SP in the model), two models are found to be equally good, one of which corresponds to an effect of WF (the other one corresponds to a quadratic effect of the age of acquisition); therefore, not taking into account SP leads to the impression that WF influences FR performance. In the end, the best model is one that does not include WF but includes SP as a predictor (a model that includes both WF and SP has a less good fit than the one with only SP as a predictor). We interpret this as a validation of our view, or at least of the fact that distributional discrepancies can be captured by a variable (here, SP) that can explain FR performance above and beyond WF — while the contrary is not true: WF does not explain FR performance above and beyond SP.

Let us consider now how an explanation of FR performance in terms of Bayesian surprise accounts for the extant WF effect results. We start with the mixed list (within-subjects design) paradigm, where positive, negative, and no relation was found between WF and FR performance. All such results are easy to explain in terms of SP: the only important matter in all three cases is for which words a high SP value is obtained. This depends on where in the WF range the distributional discrepancies are situated. If high SP values are obtained for words of both low WF (LF) and high WF (HF), then no relation will be found, while if high SP values are obtained mainly for HF (LF) words, a positive (negative) relation will be found.

A similar explanation works for the results coming from the pure list (between-subjects design) paradigm. The Bayesian surprise will be very high in participants that are presented with (the list of) HF words, because, as discussed earlier, their brains expect to be presented with medium and LF words too and that does not occur. It is reasonable to suppose that after noticing that only HF words are presented to them, the reason for this Bayesian surprise may somewhat wither. However, within the high WFs distributional discrepancies will still occur and generate Bayesian surprise / high SP values. Let us illustrate with a numerical example involving a list of three low WF words (picked out at random among the words in the second bin in Figure 2A) and one of three high WF words (picked out at random from the four bins of highest WF). Suppose the frequencies in the former are (in this order) 2.75, 13.74, 20.23, and for the latter 594.45, 927.41, 1,349.40. If for the first word of a list we use the lowest WF in the database as the lower bound for the interval involved in the computation of SP (see computation of SP), the SP values are 0.8, 1.11, 0.48 for the LF list words, and 3.22, 3.41, 3.58 for the HF list words. If one considers that in the HF list condition as the second word is presented participants’ brain realizes that only HF words are presented and thus changes its expectations accordingly, we can alter the computation of SP by using as lower bound for the interval involved in the computation of SP the WF of the first word that was presented. This yields then for the HF list words the following SP values: 3.22, 1.35, 1.79. If the words are presented such that their WF (in this order), 20.23, 13.74, 2.75, and, respectively, say, 1,349.4, 927.41, 594.45, the associated SP values are 1.73, 1.56, 0.8 for LF and, respectively, 3.22, 1.79, 2.08 for HF words. These examples, although simplistic, are representative of the dynamics involved and of the SP values that are found. As one can appreciate, SP values associated with HF words are higher than those associated with LF words, so an explanation in terms of Bayesian surprise can account for the robust positive relation between WF and FR performance found with pure lists.

Our view and the explanation proposed here only consider WF distribution discrepancies between FR experimental lists and participants’ language. There are certainly other factors that shape the cognitive system, some of which could be considered from an ecological point of view. For instance, the association between items may also explain free recall; we acknowledge this is an interesting research avenue to pursue, but our approach has nothing to say on this. Likewise, because our approach only takes into account the distribution of WF, it is silent with respect to effects on FR that rely on semantics (e.g., isolation effect) — although we are inclined to think that a furniture name in a list of animal names does trigger a Bayesian surprise signal.

We acknowledge this hypothesis should be tested in other paradigms, such as recognition, serial recall, priming, etc. We chose to concentrate on free recall because it offers a situation that allows for testing whether SP affects the performance in the absence of other considerations, without having to deal with other variables/stimuli. For instance, had we chosen to run recognition experiments, it would seem to us that the distractors used during the recognition phase also bear consequences on the recall performance and thus should somehow be taken into account; the same is true with the words recognized as learned (be they correctly or not). Indeed, all these stimuli are processed *after* the learning phase is over but *while* the recognition task is ongoing, so they can affect the recognition decisions. More work is needed to pinpoint what stimuli matter to the participant while they are carrying out the recognition task and be able to correctly make use of our index of Bayesian surprise. A similar point can be made for serial recall, where having to recall the list words in the right order may lead to conscious strategies that run parallel or counter the surprising signal that we operationalized as SP. We hope our view will spark interest among the researchers who concern themselves with free recall in particular, but also, more broadly, with memory and the interaction between memory and language. Clearly, much is left to explore and specify. Because the hypothesis of a Bayesian surprise signal in the brain is central to our view, it would be interesting that our hypothesis be tackled through neuroimaging studies.

## Data availability statement

The raw data supporting the conclusions of this article will be made available by the authors, without undue reservation.

## Ethics statement

Ethical review and approval was not required for the study on human participants in accordance with the local legislation and institutional requirements. The patients/participants provided their written informed consent to participate in this study.

## Author contributions

SM collected and analyzed the data. SM and AC contributed to the writing of the manuscript. Both authors contributed to the manuscript revision, read, and approved the submitted version.

## Footnotes

1 Actually, this is the case not only for English but for all natural languages (e.g., Piantadosi, 2014).

2 This view is reminiscent of those expressed by Jones and Macken (2015, 2018) in their work concerned with how “simple associative learning operating on the linguistic environment to which a typical child may have been exposed” predicts their performance on short-term memory measures (e.g., digit span). Our views are maybe closest to those expressed by Macken et al. (2015) when they apply their ‘embodied’ or ‘grounded’ cognition analysis to the idea of capacity-limited processing systems and conclude that performance is best understood as being not the consequence of capacity-limited processing systems but rather depending dynamically on “the particular task that must be accomplished, the nature and form of the material upon which the task must be performed, and the repertoire of skills and perceptual-motor functions possessed by the participant.” We too think it is the interaction between relational properties that preexist the experimental situation and the experimental task at hand that determines performance.

3 Given Tribus’ *surprisal* notion, we would have preferred Itti and Baldi spoke of Bayesian surpris*al*. The reader is reminded that we will use *Bayesian surprise* throughout *not* in the sense of cognitive surprise but rather in the sense of a neural signal within the brain, which does *not* necessarily result in cognitive surprise (i.e., the *awareness* that something is “not as expected”).

4 We do not assume the linguistic experience of all participants is identical, quite to the contrary. However, we had to make this simplification in order to be able to compute SP, and thus we used a WF database (although this simplification entails considering that all participants have the same linguistic experience). Making this simplification leads to SP values that are slightly different from what they would be if we had the “WF database” for each participant and could compute SP with all the possible precision. It is psychometric common knowledge that error in the measuring of a variable (here, SP) leads to an *under*estimation of its effect. Because an underestimation of the effect of SP on FR performance runs counter to our hypothesis, it only makes our approach more conservative.

5 Actually we consider all words of frequency greater than or equal to .07, as the only lower frequency in the database is zero, and it corresponds to a catchall for very rare words or awkward word forms.

6 See footnote 5.

7 Correlation coefficients between all predictors but SP were tested for significance with a degree of freedom of N-2, with N being the number of items. For correlations involving SP, because SP values of the words vary from one participant to another, the degree of freedom used was (N x P)-2, with N being the number of items and P being the number of participants. As the values on the other descriptor correlated with SP did however not vary, the significance of the correlations may be overestimated. However, because we contend that SP offers a better account of FR performance than other word descriptors (and in particular WF), it is less of a problem to commit a type-I error than to overlook correlations that may exist between SP and the other word descriptors.

8 The first four words were always the same and always presented in the same order. They were *haltère*, *astronaute*, *hachoir*, and *satellite* for list L1, and *lance*, *trombone*, *gymnaste*, and *banjo* for list L2 (cf. Appendix A for their WF and other descriptor values).

9 When searching for candidate predictors by considering models that include each such candidate and comparing those models to the reference model, any model that has a lower BIC value than the reference model is provisionally interpreted to mean that the predictor considered in that model explains a significant part of deviance of the dependent variable. The same is true for the following step when one starts from a model including many predictors (i.e., all those predictors that, when included individually in a model, seemed to explain a significant part of the deviance of the dependent variable) and prune it in order to find the model that best fits the data. Importantly, however, if there are two (or many) candidates models for the best model and one of them has i) more predictors than the other(s) and ii) a lower BIC value, it will be deemed to be the best model *only if* there is a difference of at least two points between this model’s BIC and that of the other, simpler model(s), as a difference of two points in BIC value (or a Bayes factor of more than 3) is considered the threshold for assuming there is “positive” evidence in favor of the more complex model (Raftery, 1995).

10 For instance, one of the researchers we thank for his contribution in the acknowledgments section opined that “In a random list of 30 words representative for the language, I would expect to find mostly high frequency words and a few low frequency words, exactly the opposite of what [you] expect”.

## Appendix A

**Table:** Experimental list words used in Experiment 1 and Experiment 2.

L1 Words	English word	WFA	AoA	CFam	WFC	PhN	Anim
ail	garlic	7.97	2.95	4	7.693	2	−1
arc	bow	14.05	2.4	1.85	25.838	3	−1
astronaute	astronaut	0.14	3.5	1.85	5.701	8	1
batterie	drum set	9.73	3.35	2.55	2.213	5	−1
bonnet	cap	14.66	1.95	2.95	37.805	4	−1
brouette	wheelbarrow	5.14	2.3	3.15	8.131	5	−1
caméra	camera	4.39	3.1	3.1	15.007	6	−1
cartouche	cartridge	2.91	3	2.6	0.758	6	−1
cercle	circle	42.43	2.6	3.4	31.159	5	−1
chignon	bun	11.76	2.7	3.05	1.449	4	−1
chronomètre	stop watch	2.09	3.4	2.75	5.353	9	−1
diable	devil	51.01	2.6	1.6	105.515	5	1
fée	fairy	6.62	1.45	1.8	92.831	2	1
globe	globe	8.58	3.35	3.3	8.758	4	−1
groupe	groupe	85.88	2.6	3.25	325.886	4	−1
hachoir	chopper	0.61	4	1.75	0.008	5	−1
haltère	barbell	0.07	4.2	2.35	0.287	5	−1
jambon	ham	11.01	1.8	3.6	25.519	4	−1
léopard	leopard	2.57	3.1	2.45	23.344	6	1
magnétophone	tape recorder	3.24	3.6	3.15	8.831	9	–1
marelle	hopscotch	1.76	2.1	2.8	7.825	5	–1
médicament	medicine	4.12	2.3	3.4	19.899	8	–1
mur	wall	172.57	1.45	3.2	179.634	3	–1
natte	plait	4.26	2	4	3.614	3	–1
orgue	organ	5.41	3.7	2.2	2.924	3	–1
plongeur	diver	1.69	3.15	1.85	2.542	6	1
pont	bridge	74.59	2.15	3.2	87.421	2	–1
pot	jar	32.3	1.3	4.05	117.76	2	–1
punaise	tack	1.76	3.05	3.65	1.204	5	–1
reine	queen	30	1.9	1.55	80.814	3	1
satellite	satellite	0.81	4.6	1.7	17.557	7	–1
tank	tank	1.89	3.95	1.9	0.004	3	–1
taureau	bull	10	2.3	2.95	22.498	4	1
tiroir	drawer	26.22	2.3	4.5	17.779	6	–1
torchon	dish towel	7.23	2.55	4.75	3.568	5	–1
trèfle	clover	4.19	2.45	3.35	11.732	5	–1

**Table:**

L2 Words	English word	WFA	AoA	CFam	WFC	PhN	Anim
agenda	diary	5.41	3.55	4.25	1.952	5	−1
anneaux	rings	7.97	3.2	2.45	11.077	3	−1
banjo	banjo	0.81	4.35	1.75	0.347	5	−1
bouée	lifebuoy	4.59	1.7	2.75	5.934	3	−1
bulle	bubble	6.62	1.75	2.95	40.412	3	−1
cadeau	present	32.77	1.35	4.2	91.694	4	−1
cible	dart board	8.65	3.25	2	8.545	4	−1
clavier	keyboard	3.24	3.35	3	5.236	6	−1
crochet	hook	9.8	3.15	1.7	6.382	5	−1
croix	cross	71.62	2.25	2.8	30.661	4	−1
esquimau	choc-ice	0.88	2.95	4.05	9.869	6	1
évier	kitchen sink	11.35	2.8	4.8	5.653	4	−1
garçon	boy	186.96	1.4	3.9	375.794	5	1
gymnaste	gymnast	0.61	3.75	2.2	0.592	7	1
hérisson	hedgehog	1.76	2.4	3.1	48.567	5	1
île	island	83.58	2.8	2.35	141.161	2	−1
lance	spear	0.14	3.45	1.4	17.898	3	−1
lavabo	washstand	13.85	2.25	4.85	11.109	6	−1
limace	slug	4.05	2.35	3.25	3.593	5	1
masque	mask	28.45	2.3	2.5	29.199	4	–1
moustache	mustache	28.92	1.8	2.85	23.478	6	1
note	note	39.32	2.65	2.9	68.9275	3	–1
oie	goose	5.2	2.3	3.3	53.952	2	1
os	bone	49.73	2.55	3.6	74.92	2	–1
palme	flippers	2.91	3.25	2.25	3.005	–4	–1
pelle	shovel	11.35	1.9	3.05	24.123	3	–1
pelote	ball of wool	4.19	2.65	2.65	7.408	5	–1
pendule	clock	9.86	2.4	3.85	18.599	5	–1
poireau	leek	0.88	2.9	4	12.182	5	–1
quille	bowling pin	2.57	2.7	2.3	9.738	3	–1
radeau	raft	4.32	3.25	1.5	13.507	4	–1
seau	bucket	14.73	1.8	3.85	31.856	2	–1
trombone	paper clip	0.54	3.75	3.8	3.905	6	–1
urne	urn	1.96	3.85	3.25	1.029	3	–1
ventilateur	fan	2.09	3.55	2.5	2.301	9	–1

## Appendix B

**Table:** The extremely rare words used in Experiment 2 are:

Word (in French)	English word
alunite	alunite
ammonite	ammonite
cornaline	cornaline
coxa	coxa
exuvie	exuviae
forficule	forficula
gneiss	gneiss
jaspe	jasper
lutite	lutite
ocelle	ocellus
odonate	odonata
ophiolite	ophiolite
patella	patella
ptérostygma	pterostigma
smilodon	smilodon
trilobite	trilobite
